# ABA-CsABI5-CsCalS11 module upregulates Callose deposition of citrus infected with *Candidatus Liberibacter asiaticus*

**DOI:** 10.1093/hr/uhad276

**Published:** 2023-12-23

**Authors:** Lixiao Yao, Xingru Guo, Juan Su, Qingwen Zhang, Mengyao Lian, Hao Xue, Qiang Li, Yongrui He, Xiuping Zou, Zhen Song, Shanchun Chen

**Affiliations:** National Citrus Engineering Technology Research Center, Citrus Research Institute, Southwest University, Beibei, Chongqing 400712, China; National Citrus Engineering Technology Research Center, Citrus Research Institute, Southwest University, Beibei, Chongqing 400712, China; National Citrus Engineering Technology Research Center, Citrus Research Institute, Southwest University, Beibei, Chongqing 400712, China; National Citrus Engineering Technology Research Center, Citrus Research Institute, Southwest University, Beibei, Chongqing 400712, China; National Citrus Engineering Technology Research Center, Citrus Research Institute, Southwest University, Beibei, Chongqing 400712, China; National Citrus Engineering Technology Research Center, Citrus Research Institute, Southwest University, Beibei, Chongqing 400712, China; National Citrus Engineering Technology Research Center, Citrus Research Institute, Southwest University, Beibei, Chongqing 400712, China; National Citrus Engineering Technology Research Center, Citrus Research Institute, Southwest University, Beibei, Chongqing 400712, China; National Citrus Engineering Technology Research Center, Citrus Research Institute, Southwest University, Beibei, Chongqing 400712, China; National Citrus Engineering Technology Research Center, Citrus Research Institute, Southwest University, Beibei, Chongqing 400712, China; National Citrus Engineering Technology Research Center, Citrus Research Institute, Southwest University, Beibei, Chongqing 400712, China

## Abstract

Huanglongbing (HLB) primarily caused by *Candidatus* Liberibacter asiaticus (*C*Las) has been threatening citrus production globally. Under HLB conditions, an excessive accumulation of the polysaccharide callose in citrus phloem occurs, leading to phloem blockage and starch accumulation in leaves. The callose production is controlled by callose synthases (CalS), which have multiple members within plants. However, the knowledge of callose production in the citrus upon infection with *C*Las is limited. In this study, we firstly identified 11 *CalSs* in the *Citrus sinensis* genome through bioinformatics and found the expression pattern of *CsCalS11* exhibited a positive correlation with callose deposition in *C*Las-infected leaves (correlation coefficient of 0.77, *P* ≤ 0.05). Knockdown of *CsCalS11* resulted in a reduction of callose deposition and starch accumulation in *C*Las-infected citrus. Interestingly, we observed significantly higher concentrations of abscisic acid (ABA) in HLB-infected citrus leaves compared to uninfected ones. Furthermore, the expressions of *CsABI5*, *CsPYR*, and *CsSnRK2* in the ABA pathway substantially increased in citrus leaves upon *C*Las infection. Additionally, the expression of *CsCalS11* was significantly upregulated in citrus leaves following the application of exogenous ABA. We confirmed that CsABI5, a pivotal component of the ABA signaling pathway, regulates *CsCalS11* expression by binding to its promoter using yeast one-hybrid assay, dual luciferase assay, and transient expression in citrus leaves. In conclusion, our findings strongly suggest that the CsABI5-CsCalS11 module plays a crucial role in regulating callose deposition through the ABA signaling pathway during *C*Las infection. The results also revealed new function of the ABA signaling pathway in plants under biotic stress.

## Introduction

Huanglongbing (HLB) represents one of the most devastating diseases of citrus worldwide [1], decimating the citrus industry in over 60 citrus-growing countries and regions (https://www.cabi.org). Since the disease was first reported in Florida, the United States in 2005, the citrus production in the state has decreased from 174.8 million boxes in 2005–2006 to 45.1 million boxes in 2021–2022 (https://www.nass.usda.gov). In Ganzhou, Jiangxi province, China, around 50 million affected trees were removed and destroyed from 2013 to 2018 due to an outbreak of HLB [2]. The causal pathogen of HLB are three species of phloem-limited, non-cultured, and Gram-negative α-Proteobacteria, namely *Candidatus* Liberibacter asiaticus (*C*Las), *Candidatus* L. africanus L. americanus (*C*Lam), and *Candidatus* (*C*Laf). Among them, *C*Las is the primary pathogen of HLB and is predominantly distributed through the Asian citrus psyllid, *Diaphorina citri*, and grafting with diseased scion [1, 3]. *C*Las is capable of infecting almost all commercial citrus species and scions, and still now, no resistant cultivars have been identified.

HLB symptoms in *C*Las-infected plants predominantly comprise chlorosis and asymmetrical blotchy mottling leaves, “red-nose” fruits, and rotted fibrous roots [1, 3, 4]. A growing number of research strongly suggests that the phloem transport malfunction in HLB-affected citrus is responsible for these disease symptoms. The dysfunctional phloem obstructs the transport of photosynthetic products from source to sink, resulting in starch accumulation in the leaves and nutrient deficiency in the roots [5]. This eventually leads to HLB symptoms and even plant death [6, 7]. Callose, a 1–3 linked β-glucan polymer, is a crucial contributor to phloem dysfunction in citrus affected by HLB. In symptomatic citrus plants, excessive amounts of callose deposits are visible around the sieve pore, which is usually accompanied by phloem collapse and systemic cell death [8]. Numerous callose accumulation can be also observed in symptomless leaves affected by HLB [9], while phloem cell collapse is not visible [6]. Callose deposition serves not only as a symptom of HLB, but also as an indicator of sensitivity and resistance to the disease. HLB-tolerant citrus, with lower levels of callose deposition and starch accumulation compared to susceptible citrus, can regenerate new phloem to mitigate obstacles, maintain vascular function, and sustain new shoot production [6, 10–12]. Furthermore, gene-overexpressed susceptible citrus with *SAMT1* and *NPR1* demonstrated a significant reduction in callose accumulation upon *C*Las infection and exhibited mild symptoms [13].

Callose synthase (CalS), also known as β-1,3-glucan synthases (GSL), directly catalyze callose biosynthesis utilizing UDP-glucose as substrate. CalS members have been identified and analyzed in various plants, including model plant Arabidopsis, dicot plants, and monocot plants [14–16]. These CalSs catalyzed callose biosynthesis in phloem, cell wall, cell plate, pollen tube, and other tissues and positions, playing an important role in systematic transportation, intercellular permeability, and plant protection from biotic and abiotic stresses [17]. Arabidopsis *AtCalS12* and its homologous *TaGSL22* of wheat were significantly increased by infection of fungal pathogens. Overexpression of *AtCalS12* (*PMR4*) enhanced callose deposition in the cell wall and conferred complete resistance to both the non-adapted and the virulent powdery mildew pathogens in Arabidopsis and barley [18, 19], while knockdown or knockout of *CalS* in plants could decrease callose accumulation and increase susceptibility of pathogens. Silencing of *AtCalS12* ortholog in wheat and barley increased cell wall penetration by *Blumeria graminis* [20, 21]. T-DNA insertion within *AtCalS10* caused high susceptibility to *Pseudomonas syringae* in Arabidopsis, and reduced expression of *ClCalS1* weakens resistance to *Xanthomonas citri* in lemon [9, 22]. The Atcals7ko mutation leads to increased susceptibility to Chrysanthemum Yellows phytoplasma [23].

The expression of various *CsCalSs* was modified in citrus trees affected by HLB in comparison to the healthy ones [12, 24, 25]. However, it remains unclear which key *CsCalS* enhances callose deposition in citrus phloem under HLB stress and its regulation mechanism. Here, eleven *CsCalSs* were screened and identified in the citrus genome. Among the *CsCalS* family, *CsCalS11* had been demonstrated to possess significantly higher expression in leaves of healthy citrus and was greatly upregulated in response to HLB stress. We analyzed the relationship between the expression of *CsCalS11* and the callose deposition during *C*Las infection and also identified the transcriptional factor CsABI5 that regulated *CsCalS11*. As a result, the molecular mechanism of callose deposition regulated by *CsCalS11* through the ABA pathway under HLB stress was unveiled.

## Results

### Identification of callose synthase genes of *Citrus sinensis*

**Figure 1:**
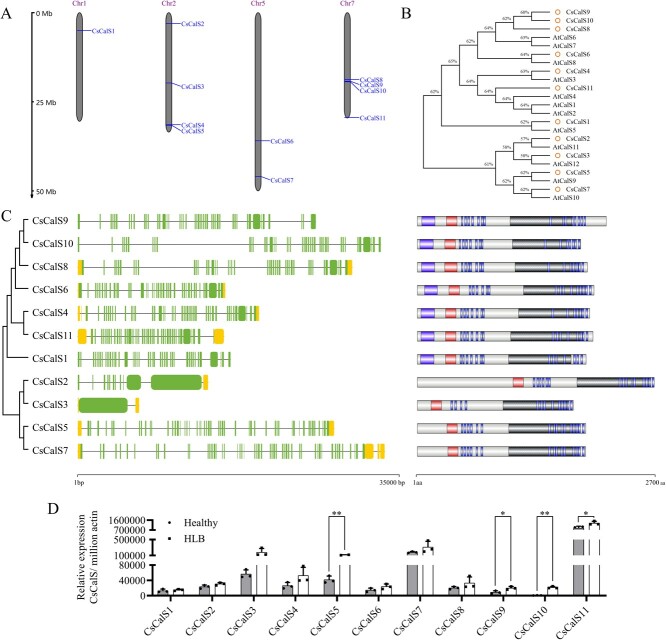
The analysis of *CsCalSs* in *Citrus sinensis*. A: Chromosomal localization of *CsCalSs*. The scale size of the chromosome is labeled on the left. The chromosome number was denoted at the top of each chromosome. The gene location was illustrated with a line and its name was on the right side of the chromosome. Chr, chromosome. B: Phylogenetic relationships of CalS proteins from *Arabidopsis thaliana* and *C. cinensis* using MEGA. AtCalS1 (AT1G05570.1), AtCalS2 (AT2G31960.1), AtCalS3 (AT5G13000.1), AtCalS4 (AT5G36870.1), AtCalS5 (AT2G13680.1), AtCalS6 (AT3G59100.1), AtCalS7 (AT1G06490.1), AtCalS8 (AT3G14570.1), AtCalS9 (AT3G07160.2), AtCalS10 (AT2G36850.1), AtCalS11 (AT4G04970.1), AtCalS12 (AT4G03550.1), and CsCalS1–12. C: Gene structure and protein domain structure of *CsCalSs*. The genomic structure of *CsCalSs* was analyzed using TBtools: Intron was shown with a horizontal line; Extron was expressed through a box. The conserved domain and transmembrane region were analyzed using the SMART tool. Vta1 domains, FKS1 domains, glucan synthase domains, and transmembrane regions were in in different colour rectangles. D: The RT-qPCR analysis of *CsCalS*s in leaves of healthy and HLB-affected “Wanjincheng” (*C. sinensis*) in the greenhouse through 2^−ΔCt^ analysis method. ^*^P ≤ 0.05; ^**^P ≤ 0.01.

Eleven *CsCalSs* were identified through screening the genome of *Citrus sinensis* V3.0 from CPBD (http://citrus.hzau.edu.cn), *C. sinensis* V1.1 of JGI (https://phytozome-next.jgi.doe.gov), and NCBI database (https://www.ncbi.nlm.nih.gov) (Supplemental Table S1). They were located on chromosomes 1, 2, 5, and 7, and their nomenclature was assigned based on their positional order as *CsCalS1-CsCalS11* (Figure 1A). Seven CsCalSs indicated a one-to-one orthologue relationship with Arabidopsis AtCalSs. In contrast, three CsCalSs (CsCalS8–10) demonstrated high homology to AtCalS6 and AtCalS7, while CsCalS11 showed homology to AtCalS2, AtCalS1, and AtCalS4 (Figure 1B). Notably, each *CsCalS* was found to encode a protein possessing one KFS1 domain, one Glucan synthase domain, and over ten transmembrane regions. In addition, some of these CsCalSs exhibited an additional conserved functional domain Vta1 in the N-terminal protein sequence, except for CsCalS2, CsCalS3, CsCalS5, and CsCalS7 (Figure 1C, Supplemental Table S2).

The relative expressions of *CsCalSs* were analyzed in healthy and HLB-affected leaves of “Wanjincheng” (*C. sinensis*) in a greenhouse. *CsCalS5*, *CsCalS9*, *CsCalS10,* and *CsCalS11* were significantly up-regulated under HLB stress (Figure 1D). Similarly, the expression of *CsCalS9* and *CsCalS11* was also dramatically increased in HLB-affected leaves of “Chandler” pummelo (*C. grandis*) and “Shatangju” (*C. reticulata*) in field (Supplemental Fig S1). In addition, *CsCalS11* expression level was higher than *CsCalS9* in *C*Las-free leaves of the above three citrus varieties, which maybe have more important role in plant growth. Therefore, *CsCalS11* was chosen for further research.

### *CsCalS11* expression had a positive correlation with callose deposition under HLB stress

**Figure 2:**
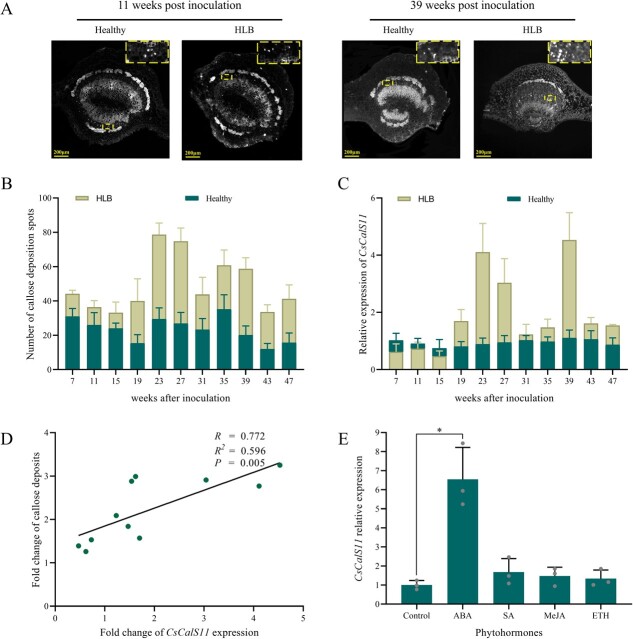
The *CsCalS11* expression was positively related to callose deposition and ABA. A: Callose deposition at 11 weeks and 39 weeks post-inoculation. The number of callose deposition (B) and relative expression of *CsCalS11* (C) were measured in HLB-affected *Citrus sinensis* and healthy ones every 4 weeks. D: A positive correlation between *CsCalS11* expression and callose deposition obtained with SPSS software (*Pearson R* = 0.772). E: The relative expression of *CsCalS11* under 100 μmol·L^−1^ abscisic acid (ABA), 10 μmol·L^−1^ salicylic acid (SA), 100 μmol·L^−1^ methyl jasmonate (MeJA), and 10 μmol·L^−1^ ethephon (ETH) for 48 hours. ^*^P ≤ 0.05.

To analysis of the relationship between *CsCalS11* expression and callose deposition in response to HLB stress, one-year-old “Wanjincheng” seedlings in the greenhouse were subjected to *C*Las inoculation through leaf disc grafting. At seven week post-inoculation (wpi), *C*Las was detected to be positive for the first time. Hence, the relative expression of *CsCalS11* was quantified by RT-qPCR between HLB plants and healthy ones every 4 weeks since 7 wpi, along with counting callose spots. No significant variations in *CsCalS11* expression or callose deposits were observed between HLB and healthy leaves from 7 to 15 wpi. However, starting at 19 wpi, a significant increase in both the *CsCalS11* expression and callose deposits was observed in the HLB citrus. At 39 wpi, the fold change in *CsCalS11* expression and callose deposits in HLB citrus reached a peak, with 4.53- and 3.25-fold expression respectively, compared with those in the healthy control (Figure 2A-2C). Furthermore, statistical analysis results revealed a positive correlation between the *CsCalS11* expression and callose deposition under HLB stress, with a *Pearson* correlation coefficient of 0.77 (*P* ≤ 0.05) (Figure 2D).

To compare the expression of *CsCalS11* in citrus leaves treated with different phytohormones, RT-qPCR was performed. The results showed that *CsCalS11* was significantly induced by 100 μmol∙L^−1^ abscisic acid up to 6.54 times higher than in the control. Its expression could not be changed by 10 μmol∙L^−1^ salicylic acid, 100 μmo∙L^−1^ methyl jasmonate, and 10 μmol∙L^−1^ ethephon (Figure 2E).

### *CsCalS11* transient overexpression enhanced callose deposition

*CsCalS11* was located on chromosome 7, comprising 42 exons and 41 introns. The open reading frame (ORF) of *CsCalS11* was 5907 bp in length, encoding a protein of 1968 aa with a molecular weight of 226.64 kDa. The putative protein sequence of *CsCalS11* contained three conserved motifs (Vta1, FKS1, and Glucan-synthase) and 15 transmembrane regions (Supplemental Table S2). CsCalS11 had high identities with AtCalS2 (83%), AtCalS1 (83%), and AtCalS3 (82%).

**Figure 3:**
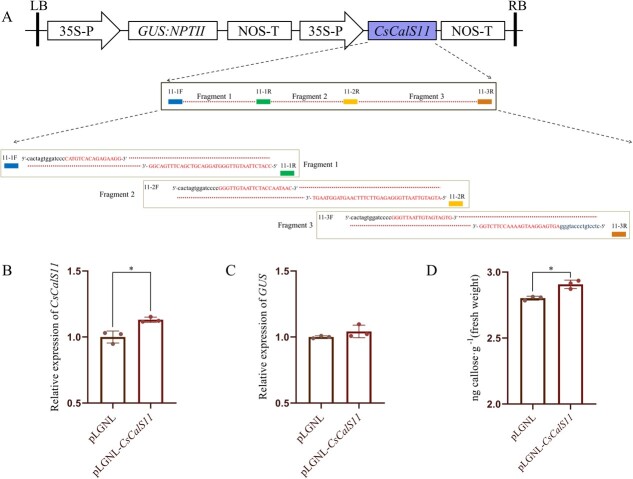
Transient expression of *CsCalS11* in citrus leaves. A: Schematic representation of the *CsCalS11* overexpressing vector based on the pLGNL binary plasmid employing the In-fusion strategy. 11–3F/11–3R, 11-2F/11-2R, and 11-1F/11-1R were primer pairs for cloning fragments 3, 2, and 1 of the *CsCalS11* respectively. pLGNL, 11–3F, and 11-2F include the *Sma*I cleavage site CCCGGG. The primer sequences from *CsCalS11* are represented in capital letters and lowercase sequences from pLGNL in black letters. The expression of *CsCalS11* (B) and *GUS* (C) and callose content (D) were quanlitified in transiently overexpressed citrus. The experiments were repeated for three times with the similar results. ^*^: *P* ≤ 0.05.

*CsCalS11* was difficultly cloned into the overexpression plasmid pLGNL in *Escherichia coli* at one time for its long sequence with nearly 6000 bp, which was larger than most plant genes. The pLGNL-*CsCalS11* was successfully constructed through cloning fragment 3 (2477 bp), fragment 2 (1689 bp), fragment 1 (1693 bp) onto vector pLGNL (Figure 3A). For confirming the function of *CsCalS11* in callose biosynthesis, it was transiently overexpressed in “Wanjincheng” (*C. sinensis*) leaves. As expected, the expression of *CsCalS11* was significantly up-regulated and the reference gene *GUS* was not changed. Furthermore, callose content were significantly increased in transiently overexpressed leaves in three separate experiments (Figure 3).

### *CsCalS11* knock-down decreased callose deposition in sweet orange

**Figure 4:**
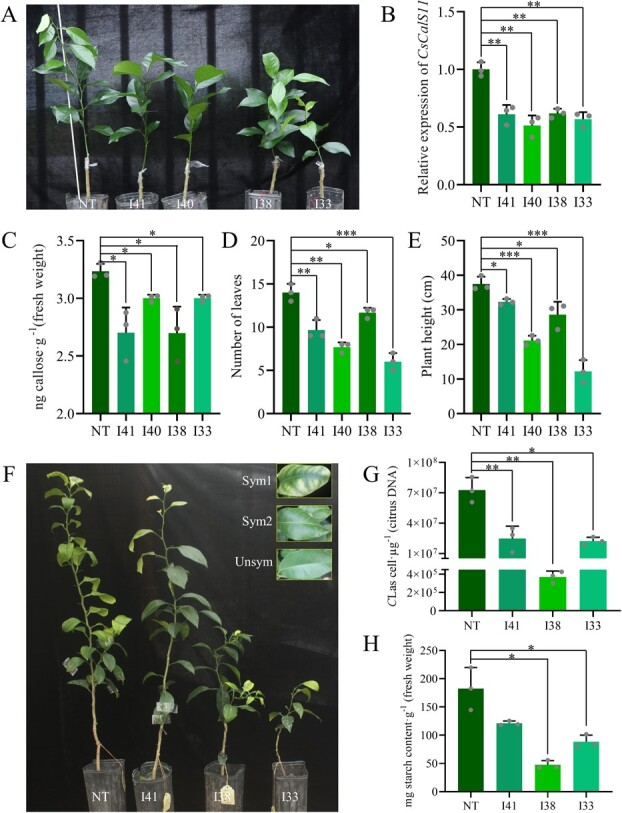
Evaluation of *CsCalS11*-RNAi citrus strains under normal conditions (A-E) and Huanglongbing (HLB) stress (F-H). A: Four *CsCalS11*-RNAi citrus strains and non-transgenic control (NT). *CsCalS11* relative expression (B), callose concentration (C), leaf number (D), and plant weight (E) of *CsCalS11*-RNAi strains (3 plantlets for each strain). F: HLB symptoms observation. Sym1 shows leaf blotch chlorosis; Sym2 shows the burst leaf vein; Unsym means asymptomatic leaves. *C*Las (G) and starch (H) contents in leaves of *CsCalS11*-RNAi seedlings affected by HLB. ^*^: *P* ≤ 0.05; ^**^: *P* ≤ 0.01; ^***^P ≤ 0.001.

Four *CsCalS11*-RNAi plant strains (I-33, I-38, I40, and I-41) were generated by *Agrobacterium tumefaciens* carrying the vector pLGNL-*CsCalS11*-RNAi (Figure 4A). The propagation was subsequently carried out using the grafting technique, and three plantlets were obtained from each of the strains. Compared to the non-transgenic controls, *CsCalS11* expression was significantly down-regulated in the range of 38.02% to 48.72% through RNA interference (Figure 4B). The callose content in leaves of *CsCalS11*-RNAi citrus seedlings was significantly decreased, from 3.23 ± 0.05 ng∙g^−1^ in non-transgenic ones down to 2.70 ± 0.16 ng∙g^−1^ (Figure 4C). Compared with the non-transgenic control, the average value of leaf number and plant height were reduced in the *CsCalS11*-RNAi scions, being down-regulated with 16.67% to 57.14% and 13.94% to 67.41%, respectively (Figure 4D-4E). At 10 months after being grafted with *C*Las-infected leaf discs, the symptomatic leaves of the *CsCalS11*-RNAi plants only exhibited chlorosis, while the non-transgenic controls presented serious symptoms with both leaf vein burst and chlorosis (Figure 4F). The callose concentration in the leaves of *CsCalS11*-RNAi plants was further reduced by 31.04% to 43.70% compared with that of the non-transgenic citrus. Furthermore, the titer of *C*Las was extremely significantly decreased in the *CsCalS11*-RNAi plants, especially the bacteria population in I-38 was 0.51% of ones in non-transgenic citrus (Figure 4G). Meanwhile, the starch content in the *CsCalS11*-RNAi plants infected with *C*Las decreased by 33.59% to 73.95% compared with the non-transgene control, with I-38 owning the lowest starch 47.50 mg∙g^−1^ (fresh weight) (Figure 4H).

### *CsCalS11* transcription was activated by CsABI5 via directly binding on its promoter

The *CsCalS11* promoter (CsCalS11p), 1500-bp upstream of the starting codon, were analyzed with PlantCARE software and found several hormone-responsive *cis*-elements in the promoter sequence. They were an ABRE motif (involved in the ABA responsiveness), a TCA-element (involved in salicylic acid responsiveness), and two GARE-motif (involved in gibberellin responsiveness). Moreover, *cis*-acting elements involved in abiotic stress responsiveness were identified, including a WUN motif (involved in wound responsiveness) and MBS (involved in drought-inducibility) (Supplemental Table S3).

To screen candidate proteins that might directly regulate *CsCalS11*, a cDNA library was constructed using equally mixed RNA extracted from leaves of both HLB “Wanjincheng” and healthy ones. The titer of the cDNA library is 9.26 × 10^7^ cfu∙mL^−1^, and the length of 80% cDNA insertion was more than 750 bp. The cDNA library was then subjected to yeast one-hybrid assay with pAbAi-CsCalS11p as bait. Twenty-four candidate proteins were identified, which showed potential binding ability with the *CsCalS11* promotor (Supplemental Fig. S2 and Supplemental Table S5).

**Figure 5:**
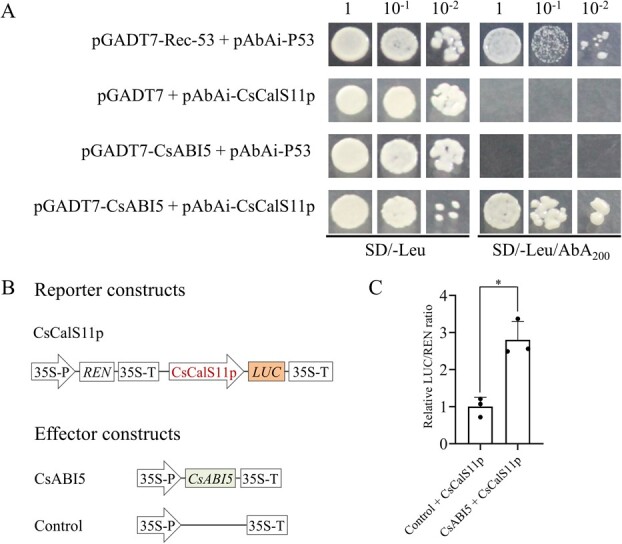
The CsCalS11p was bound and activated by CsABI5 in yeast and tobacco. A: Physical interactions of CsABI5 with CsCalS11p in yeast one hybrid (Y1H) assays. The pGADT7-Rec-53 was introduced into yeast cells carrying pAbAi-P53 as a positive control. The empty vector pGADT7 was included as a negative control. Another negative control was yeast containing pGADT7-CsABI5 and pAbAi-P53. AbA_200_ means 200 ng·nL^−1^ Aureobasidin A. B: Reporter and effector constructs for dual LUC assays. LUC: firefly luciferase, REN: Renilla luciferase, 35S-P: CaMV35S promoter, 35S-T: CaMV35S terminator. CsCalS11p::*LUC* was constructed based on pGreenII 0800-LUC. The control effector construct was from the pLGNL plasmid. C: Dual luciferase activity assay performed by transient expression in *Nicotiana benthamiana* leaves. ^*^P ≤ 0.05.

To further identify the binding and activating ability, the open reading frames (ORFs) of a transcriptional factor *CsABI5* in the ABA signal pathway was cloned into the pGADT7 vector. Subsequently, one-on-one bait/prey interactions were conducted and revealed that CsABI5 could bind to the *CsCalS11* promoter (Figure 5A). At the same time, a dual-luciferase expression assay was conducted in tobacco leaves. The results demonstrated that CsABI5 could enhance the transcription of the luciferase gene driven by the promoter of *CsCalS11* to 2.81 times, with a significant increase in luciferase activity using REN as a reference (Figure 5C).

**Figure 6:**
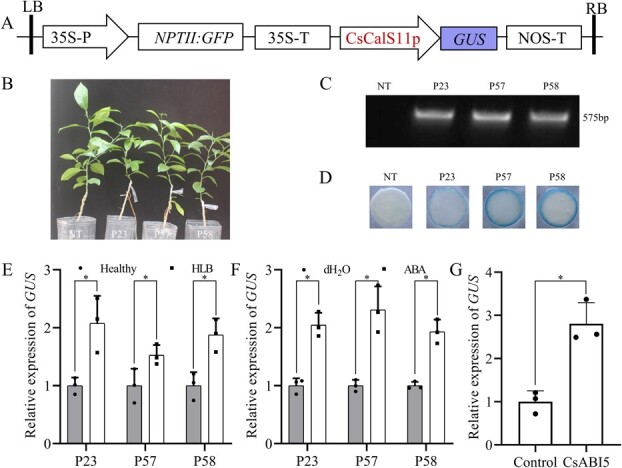
The *CsCalS11* promoter (CsCalS11p) was activated under conditions of Huanglongbing (HLB) stress, exogenous abscisic acid (ABA) exposure and transient overexpression of *CsABI5*. A: Schematic representation of the CsCalS11p::*GUS* constructs T-DNA of the pGNGM1300 binary vector. B: The transgenic seedlings with CsCalS11p::*GUS*. C: PCR analysis with primer pair GUS-F/GUF-R using leaf DNA from (B) seedlings. D: GUS staining of leaf discs from (B) seedlings. E: The relative expression of *GUS* in CsCalS11p::*GUS* seedlings affected by HLB compared with the healthy one. F: The relative expression of *GUS* in CsCalS11p::*GUS* leaves treated with 100 μmol·L^−1^ ABA compared with H_2_O. G: *GUS* expression in the citrus leaves transformed with CsCalS11p::*GUS* through transient expression of *CsABI5*. NT: non-transgene seedling; P23/P57/P58: three citrus strains transformed with CsCalS11p::*GUS*.

For examining CsCalS11p activation in citrus, the promoter was inserted upstream of the reporter gene *GUS* in the pGNGM1300 vector (Figure 6A). Through *A. tumefaciens* transformation, three independent transgenic citrus lines containing CsCalS11p::*GUS* (P23, P57, and P58) were generated without significant differences to those non-transgene plants in vegetative growth patterns (Figure 6B-6D). After 42 weeks post-infection with *C*Las, the *GUS* expression in the transgenic citrus lines was increased compared with that of the healthy control (Figure 6E). On the other way, the expression of *GUS* was significantly up-regulated to 2.09, 2.30, and 1.93 times respectively in leaves of P23, P57, and P58 treated with 100 μmol·L^−1^ ABA (Figure 6F). When CsABI5 was transiently expressed in P23 leaves transformed with CsCalS11p::*GUS*. The overexpression of the transcriptional factor significantly upregulated the expression of *GUS* driven by the *CsCalS11* promoter to 1.86 times (Figure 6D). Therefore, these findings suggested that the transcription of *CsCalS11* could be activated by *C*Las, ABA, and CsABI5 *in vivo*.

### ABA concentration and genes in the ABA signal pathway were induced by *C*Las

**Figure 7:**
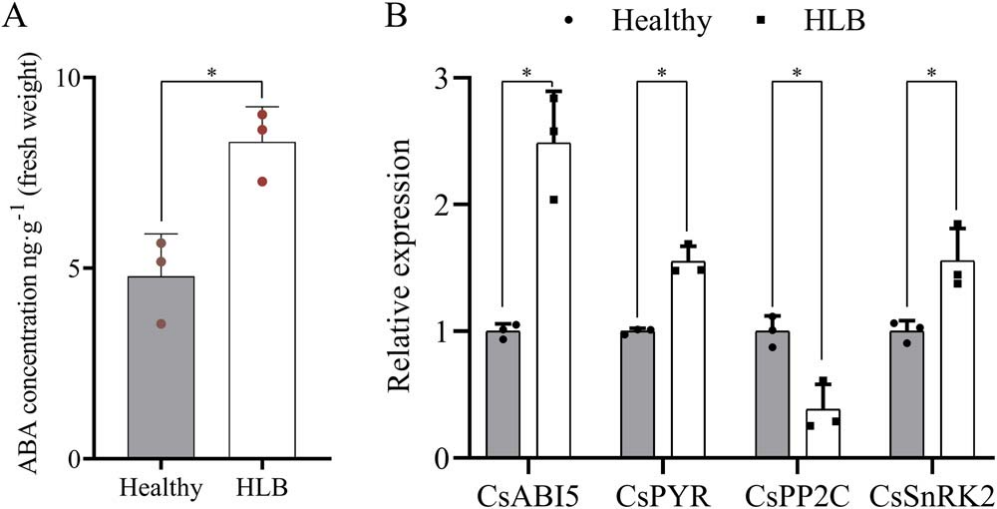
The concentration of abscisic acid (ABA) and the expression of genes in its signal pathway in healthy and Huanglongbing (HLB) citrus. A: ABA concentration. B: Fold change of *CsABI5*, *CsPYR*, *CsPP2C*, and *CsSnRK2* expression in citrus under HLB stress. ^*^P ≤ 0.05.

The results of exogenous phytohormone experiments showed that the expression of *CsCalS11* was positively induced by ABA (Figure 2E and6F). Therefore, we investigated the concentration of ABA and the expression of genes related to the ABA signaling pathway in citrus leaves under HLB stress. It was found that ABA concentration presented a significant increase to 8.31 ng·g^−1^ in HLB citrus, 1.73 times greater than the phytohormone in healthy one (Figure 7A). Moreover, some important genes in the ABA signal pathway were up-regulated in citrus affected by HLB. The expression of a PYR/PYL/RCAR-like receptor binding with ABA (*CsPYR*), protein kinase *CsSnRK2*, and *CsABI5* was significantly increased in citrus leaves affected by HLB compared to the healthy leaves. A negative regulator in the ABA pathway, *CsPP2C*, was down-regulated in HLB-affected citrus. These results indicated that ABA accumulation and its signal pathway could be induced in citrus by *C*Las infection.

## Discussion

### Multiple *CsCalSs* were stimulated in response to *C*Las

Callose strengthens the cell wall and regulates the permeability of intercellular junction via plasmodesmata. Therefore, the biosynthesis of callose to combat infections induced by fungal, viral, and bacterial pathogens has garnered considerable attention. AtCalS12 is the main biosynthetic enzyme responsible for the callose deposition at the invasion site of fungal pathogens, and its regulation hinges on salicylic acid and NPR1 dependence [26, 27]. Its homologous *TaGSL22* in wheat is the only CalS gene induced by *Blumeria graminis* [20]. Additionally, *AtCalS1*, *AtCalS5*, A*tCalS9,* and *AtCalS10* can be induced by *Hyaloperonospora arabidopsis* infection [27]. AsCalS1-like gene was up-regulated by *Pseudoperonospora cubensis* (Downy mildew), and AsCalS5-like and AsCalS10-like were increased by *Sphaerotheca fuliginea* (powdery mildew) in cucumber as well [28]. Otherwise, the level of AtCalS7-like and AtCalS5-like was significantly increased in Arabidopsis infected with Chrysanthemum Yellows phytoplasma and cotton fed on by aphids respectively [23, 29].

*C*Las is a phloem-restricted bacterium that is unevenly distributed throughout citrus plants. Even within the same *C*Las-positive plant, various leaves contained varying concentrations of the pathogen and some of the leaves are not colonized by the bacterium at all. These variations present significant challenges when evaluating the expression of *CsCalSs* in citrus affected by HLB. Regardless of the citrus variety and timing of *C*Las infection, the expression of AtCalS2, AtCalS7, AtCalS8, AtCalS9, and AtCalS12-like gene members were positively stimulated in response to HLB stress. AtCalS2-like and AtCalS7-like genes are potentially major regulators of callose accumulation in the HLB-affected phloem as they were significantly increased in diseased *C. sinensis*, *C. grandis*, and *C. reticulate* in both our lab and Granato’ lab [25].

### *CsCalS11* served an important role in the callose deposition during *C*Las infection

Although the expression of multiple *AtCalSs* can be regulated under biotic stresses, only a few of them have be revealed function. AtCalS12 catalyzes callose biosynthesis in papillae of the cell wall to strengthen host resistance when the plant is infected with fungal pathogens [18]. The atcals7ko mutant of Arabidopsis is sensitive to phytoplasma due to enhanced plasmodesmata permeability [23]. Little is known about *AtCalS2*, except that it physically interacts with a Phytophthora effector protein RxLR3 [30].

Several *CsCalS* genes are up-regulated in citrus leaves under HLB stress. However, the major gene contributing to callose deposition remains unclear. *CsCalS11*, homologous with *AtCalS2*, was significantly increased in the HLB-positive citrus not only in the greenhouse but also in the field, compared with the healthy trees (Figure 1D and Supplemental Fig. S1). Additionally, its fold change between *C*Las-infected and healthy leaves possessed a positive correlation with the ratio of callose deposits in the phloem of leaf midrib (Figure 2D). *CsCalS11* knock-down could significantly reduce callose deposition in citrus under HLB stress. Moreover, the marker gene *GUS* driven by the CsCalS11p promoter could be induced by *C*Las in CsCalS11p::*GUS*-transformed seedlings (Figure 6E). Thus, it could be concluded that *CsCalS11* plays a vital role in callose deposition in the phloem of *C*Las-infected citrus.

Callose in the phloem of *C*Las-infected citrus hinder the transportability of sieve elements, consequently leading to HLB symptoms [5, 6, 24]. Its accumulation is regulated by two *C*Las-secreted polypeptides in opposing ways, as the function of *C*Las-effectors has been increasingly investigated. A Sec-dependent polypeptide SECP8 of *C*Las localized in nucleus, cytoplasm, and cytoplasmic membrane and depressed callose deposition in *Nicotiana benthamiana* [31]. While the Las5315mp effector elicits significant callose deposition, cell death and starch accumulation in tobacco upon transient expression [32]. It is necessary to analyze whether these effectors can physically interact with CsCalS11 and their regulatory role in the activity of callose synthase for the future research.

### *CsCalS11* was increased by CsABI5 via ABA signal pathway

Callose biosynthesis is known to be promoted by abscisic acid (ABA) [33]. In the dormant bud of Hybrid aspen (*Populus tremula × tremuloides*), ABA can induce the expression of *CalS1* by repressing PICKLE, a chromodomain protein, and enhancing the expression of the transcription factor of SVL (short vegetative phase-like) [34]. This process was dependent on PP2C family protein ABI1 interacting with the ABA receptor PYR/PYL/RCAR [35]. In plant resistance to fungal pathogens, it is also found that exogenous ABA can induce CalS activity and promote callose deposition [33]. However, it has not been revealed which of *CalSs* would be dependent on ABA in plant resistance to biotic stress and its regulating mechanism.

In *C*Las-infected citrus leaves and fruit flavodo, a notable increase in ABA levels was observed compared with the healthy ones [36, 37]. The ABA signaling positively modulates plant resistance against various pathogens by facilitating the accumulation of callose in host plants [38]. Our study revealed that *CsCalS11* expression could be induced by exogenous ABA (Figure 2E). Moreover, the expression of marker gene *GUS* driven by the *CsCalS11* promoter was upregulated in leaves of CsCalS11p::*GUS* transformed citrus treated with ABA (Figure 6F). This suggested that *CsCalS11* can be regulated via the ABA signal pathway during *C*Las infection.

**Figure 8:**
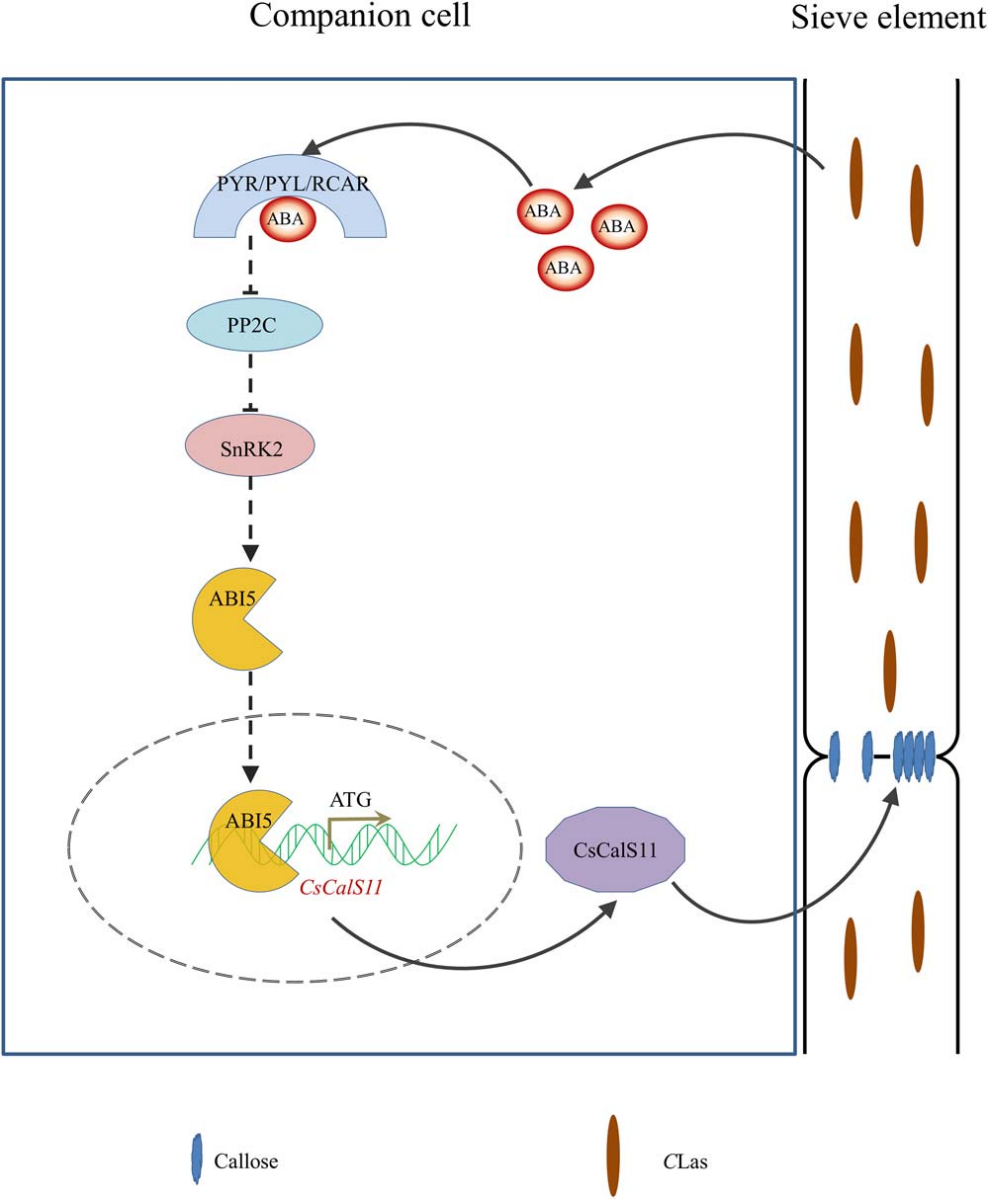
A hypothesis of callose deposition via ABA-CsABI5-CsCalS11 module during infection of *Candidatus* Liberibacter asiaticus (*C*Las).

ABI5 is a bZIP transcription factor that derives its name from the insensitivity to ABA in mutant form [39]. In response to biotic and abiotic stresses, ABA binds to its PYR/PYL/RCAR-type receptors, and the latter combines and deactivates ABA repressor PP2C. Subsequently, the SnRK2 is dissociated from PP2C and activated through self-phosphorylation, resulting in the phosphorylation and activation of ABI5 by SnRK2 [40]. ABI5 can enhance the expression of ABA-responsive genes by binding to the ABRE motif in the promoter [39–41]. In addition to preventing seed germination and post-germinative growth under unfavorable conditions, ABI5 also regulates physiological processes under abiotic stress during vegetative growth [42]. In this study, *CsABI5* was found to activate the expression of *CsCalS11* by directly binding to its promoter (Figure 5 and6G). Moreover, ABA concentration in HLB-affected leaves was significantly 1.73 times higher than that in healthy leaves (Figure 7A). *CsABI5* and other genes in ABA signal pathway were up-regulated by *C*Las infection (Figure 7B). Therefore, it is reasonable to speculate that the regulation of callose deposition in HLB citrus phloem occurs through an ABA-CsABI5-CsCalS11 module (Figure 8).

## Conclusion

This study revealed novel insights into the critical functions of *CsCalS11* in mediating callose deposition in citrus under HLB stress and presented a comprehensive regulatory model. Specifically, *C*Las infection stimulated the accumulation of ABA, which was followed by enhanced expression of the transcriptional factor *CsABI5*. *CsABI5* activated *CsCalS11* by directly binding to its promoter. The resulting upregulation of *CsCalS11* contributed to an abnormal callose deposition in the phloem.

## Material and methods

### Plant material and treatments

One-year “Wanjincheng” (*C. sinensis*) plants grafted onto “Ziyang Xiangcheng” (*C. junos*), RNA-interference plantlets, and transgenic plants with CsCalS11p::*GUS* were cultivated in a greenhouse with an average temperature of 26°C. These trees were infected with *C*Las through leaf disc grafting with restricted access, and the infection was subsequently confirmed by PCR [43]. All primers are listed in Supplemental Table S4 and synthesized in Tsingke Biotechnology Company. Leaves of *C. grandis* and *C. reticulata* were harvested from a commercial citrus grove in Guangxi province, China. Three HLB-affected citrus trees and three healthy ones were chosen for biological replicates in greenhouse and in field. Four fully mature leaves were collected per citrus and mixed for experiments.

### Identification and bioinformatics analysis of the callose synthase enzyme family in citrus

The coding DNA sequences encoding callose synthase in *C. sinensis* were retrieved from NCBI (http://www.ncbi.nlm.nih.gov) and the citrus genome databases CPBD (http://citrus.hzau.edu.cn) and Phytozome V12 (https://phytozome.jgi.doe.gov). Following the removal of redundant sequences, all putative CsCalS protein sequences were subjected to the Simple Modular Architecture Research Tool (SMART, http://smart.embl-heidelberg.de) analysis, whereby the sequences with the Glucan synthase domain, FSK1 domain, as well as N-terminal and C-terminal transmembrane regions, were considered as true CsCalS protein in citrus. The exon–intron organization was determined with TBtools [44]. Phylogenetic reconstruction was accomplished through the maximum likelihood method with MEGA V11 [45].

### RNA extraction and RT-qPCR

Total RNA was extracted from leaf tissues with an EASY spin plant RNA extraction kit (Aidlab, Beijing, China), as well as with subsequent elimination of genomic DNA using DNase (Takara, Beijing, China). First, strand cDNA was synthesized from 0.5 μg of total RNA using the 5× PrimeScript RT Master Mix (TaKaRa, Beijing, China) according to the manufacturer's instructions. qPCR was performed using ChamQ™ Universal SYBR qPCR Master Mix (Vazyme, Jiangsu, China) on CFX96™ Real-Time System (BIO-RAD, California). Amplifications were conducted in triplicate for each sample, accompanied by the appropriate negative controls, using the following conditions: 95°C for 1 minute, 40 cycles of 95°C for 15 seconds, and 60°C for 1 minutes. The housekeeping gene actin (GenBank No. GU911361.1) was applied as a reference gene for RT-qPCR analysis. All experiments were performed on three biological replicates. The relative expression values were calculated with the 2^−ΔΔCt^ method unless differently indicated.

### Phytohormone treatments

Citrus leaves were treated with exogenous phytohormones according to the description from Yang et al [46]. Mature leaves of Wanjincheng were collected and cut into leaf discs using a punch. The mixed leaf discs were divided into triangular bottles with 100 μmol∙L^−1^ abscisic acid, 10 μmol∙L^−1^ salicylic acid, 100 μmol∙L^−1^ methyl jasmonate, or 10 μmol∙L^−1^ ethephon and cultured at room temperature for 48 hours. Hormone treatments were repeated three times and leaves treated with distilled water served as controls.

### Observation of callose deposition

Leaf midribs were cut into a length of 2 to 3 mm and immersed in a 4% paraformaldehyde universal tissue fixation solution, followed by dehydration using acetone solutions and being embedded in LR White Resin according to the description from Zou *et al.* [13]. Subsequently, 0.8 μm slices were obtained from the embedded midribs through a Leica EM UC7 Ultramicrotome (Leica Microsystems, Wetzlar, Germany) and stained with 0.05% aniline blue for 5 seconds. The resulting blue-stained callose deposition in the phloem was captured by an Olympus BX51 microscope (Olympus, Tokyo, Japan) and counted with ImageJ software.

### *CsCalS11*-overexpressed plasmid construction

The overexpressed vector of *CsCalS11* was constructed in sections. Firstly, the PCR product of 5–3 fragment (5-3F/5-3R primers) was linked into pLGNL being cleaved in restriction site CCCGGG with *Sma*I to acquire the intermediate vector pLGNL-*CsCalS11*–3. Then, a 5–2 fragment (5-2F/5-2R primers) was inserted into the CCCGGG site to get pLGNL-*CsCalS11*–3-2. Finally, the overexpression vector pLGNL-CsCalS11 was obtained by inserting a 5–1 fragment (5-1F/5-1R primers).

### *CsCalS11*-RNAi plasmid construction

The *CsCalS11* RNAi vector was constructed and transformed into citrus via the method described by Yao et al [47] with a few modifications. A 398-bp fragment was amplified by PCR with CsCalS11-i3F and CsCalS11-i3R primers. The restriction enzyme sites of *Swa*I and *Xba*I were present in the CsCalS11-i3F primer, while *Asc*I and *Bam*HI were present in the CsCalS11-i3R primer. The 398-bp PCR products were digested with *Swa*I and *Asc*I to acquire forward fragments and with *Xba*I and *Bam*HI for reverse fragments. Both fragments were then inserted into the corresponding sites of the pUCRNAi plasmid, respectively. The *CsCalS11*-RNAi fragment was digested with *Kpn*I and *Sal*I from the reconstituted pUCRNAi and integrated into the pLGNL.

### Plasmid construction of CsCalS11p::*GUS*

A 1500 bp region upstream of the translation initiation codon of *CsCalS11* (Cs_ont_7g029230.1) was amplified from *C. sinensis* genomic DNA with specific primers CsCalS11P-F and CsCalS11P-R. The CsCalS11p PCR products were ligated with a pUCm-T vector for sequencing, and the correct sequence was predicted for *cis*-acting elements through PlantCARE [48]. Subsequently, the CsCalS11p fragment was digested using *Bam*HI/*Pst*I and inserted into the *Bam*HI/*Pst*I site of the pGNGM1300. The resulting recombinant vector including CsCalS11p::*GUS* was generated and transformed into the *A. tumefaciens* strain EHA105.

### Measurement of callose concentration

The callose concentrations were quantified through the application of a plant callose ELISA kit (Boshen, Jiangsu, China), according to the manufacturer’s protocols. The detection was conducted at 450 nm wavelength with JS-THERMO Varioskan Flash (Thermo, Massachusetts, USA).

### Abscisic acid (ABA) quantification

Three mature leaves were collected per line and ground into a fine powder using liquid nitrogen. The endogenous ABA content of three HLB-citrus lines and healthy lines was determined by extraction and detection with LC- MS/MS (AB Sciex TripleTOF 5600+) through multiple reaction monitoring as described previously [49].

### Starch quantification

Starch was extracted according to the protocol of the Starch Assay Kit (G-clone, Beijing, China). Briefly, the diluted starch samples were incubated with reaction buffer at 95°C for 10 min and measured at 620 nm using glucose as a standard. The starch content in fresh leaves was calculated using the formula: Starch content (mg∙g^−1^ fresh weight) = 13.51×·F, where x represents the starch concentration calculated with glucose standard, and F represents the dilution rate of the sample.

### Yeast one-hybrid assay

The yeast one-hybrid assay was conducted according to the instructions provided by the Matchmaker® Gold Yeast One-Hybrid Library Screening System (Clontech, California, USA). *CsABI5* (synthesized by Tsingke, Chongqing, China) were inserted into the prey plasmid pGADT7 through in-fusion cloning. pGADT7-*CsABI5* and the pAbAi reporter vector (“bait”) containing the *CsCalS11* promoter were co-transformed into the Y1HGold strain. The transformants were cultured on SD/-Leu/AbA_200_ medium for 3 days at 30°C.

### Dual luciferase assay

The CsCalS11p promoter was amplified using eCsCalS11p-F/R primer pairs and inserted into the pGreenII 0800-LUC plasmid after being digested with *Sal*I and *Bam*HI. This promoter was utilized to regulate the expression of the firefly luciferase gene (LUC) in pGreenII 0800-LUC, with the Renilla luciferase gene (REN) under CaMV 35S promoter serving as an internal control of the transient expression. *CsABI5* was cloned into the pLGNL vector for protein expression and served as effectors. The reconstituted vectors were separately transformed into *A. tumefaciens* strain GV3101 via the freeze–thaw method. The agroinfiltrated *N. benthamiana* leaves were grown at 25°C for 4 days and analyzed using the dual-glo® luciferase assay system (Promega, Wisconsin, USA) in a 96-well plate [50]. The LUC/REN ratio was calculated.

### Citrus transformation

The cut epicotyls were employed as explants for citrus transformation with *A. tumefaciens* following the methodology described by Zou *et al*. [13]. The transformed seedlings were firstly selected through GUS staining or green fluorescence with a LUYOR-3415RG hand-held lamp, and then confirmed by PCR with DNA as template. When the transformed citrus strains grew to 30 cm, they were propagated three to five seedlings for each strain through grafting onto the “Ziyang Xiangcheng” rootstock.

### Transient expression in plant leaves

*A. tumefaciens* strain GV3101 harboring the reconstructed vectors were injected into the citrus leaves, with a method that combined the protocols of Li *et al.* [51] and Acanda *et al*. [52]. The overnight grown GV3101 was centrifuged at 5000 rpm for 10 min and resuspended to an A_600_ of 0.8 with MMA solution (10 mmol∙L^−1^ MgCl_2_, 15 mmol∙L^−1^ 2-(*N*-morpholino) ethane sulfonic acid, 200 μmol∙L^−1^ acetosyringone, pH = 5.6). The three fully expanded leaves were subjected to surface sterilization and infiltrated until 1/3 of the leaf was saturated. Subsequently, they were placed on AIM solid medium (0.4% (w/v) MT, 0.4% (w/v) sucrose, 15 mmol∙L^−1^ 2-(*N*-morpholino) ethanesulfonic acid, 200 μmol∙L^−1^ acetosyringone, pH = 5.6) and incubated at 22°C in the dark for 6 days. Following this, the leaves were collected for RT-qPCR analysis. As a negative control, leaves infiltrated with GV3101 containing the empty pLGNL plasmid were used. This experiment was replicated three times.

### Statistical analysis

The experiments were performed with three replicates. GraphPad Prism version 8.0.2 (Graphpad Prism Inc., USA) was used for statistical analysis. All comparisons in the study were made using Student's *t*-test. *P* ≤ 0.05 was considered statistically significant, *P* ≤ 0.01 was very significant, and *P* ≤ 0.001 was extremely significant. Results were expressed as mean values ± standard deviation (SD).

## Acknowledgements

The manuscript has been revised based on feedback from Prof. Changyong Zhou at Southwest University, Prof. Yiwen Deng at CAS Center for Excellence in Molecular Plant Sciences, and peer reviewers. We express our gratitude to them. The project is financially supported by the National Key R&D Program of China (No. 2021YFD140080), Chongqing Natural Science Foundation (CSTB2023NSCQ-MSX0519), and Earmarked Funds for the China Agriculture Research System (CARS-26).

## Author contributions

L.Y., Z.S., and S.C. conceived and designed this research. X.G., J.S., Q.Z., M.L., and H.X. experimented. L.Y., Q.Z., and Y.H. analyzed the data. L.Y., Z.S., Q.L., and X.Z. wrote and modified the manuscript. All authors involved in this study read and approved the manuscript.

## Conflict of interest statement

The authors have no conflict of interest to declare.

## Data availability

The data that support the findings are available within the article and supplementary data.

## Supplementary Data

Supplementary data is available at Horticulture Research online.

## Supplementary Material

Web_Material_uhad276

## References

[1] Bové JM . Huanglongbing: a destructive, newly-emerging, century-old disease of citrus. J Plant Pathol. 2006;88:7–37

[2] Zhou C . The status of citrus Huanglongbing in China. Trop plant pathol. 2020;45:279–84

[3] da Graca JV , Douhan GW, Halbert SE. et al. Huanglongbing: an overview of a complex pathosystem ravaging the world's citrus. J Integr Plant Biol. 2016;58:373–87 26466921 10.1111/jipb.12437

[4] Braswell WE , Park JW, Stansly PA. et al. Root samples provide early and improved detection of *Candidatus* Liberibacter asiaticus in *citrus*. Sci Rep. 2020;10:16982 33046775 10.1038/s41598-020-74093-x PMC7550583

[5] Koh EJ , Zhou L, Williams DS. et al. Callose deposition in the phloem plasmodesmata and inhibition of phloem transport in citrus leaves infected with “*Candidatus* Liberibacter asiaticus”. Protoplasma. 2012;249:687–97 21874517 10.1007/s00709-011-0312-3

[6] Fan J , Chen C, Yu Q. et al. Comparative transcriptional and anatomical analyses of tolerant rough lemon and susceptible sweet orange in response to '*Candidatus* Liberibacter asiaticus' infection. Mol Plant-Microbe Interact. 2012;25:1396–407 22809274 10.1094/MPMI-06-12-0150-R

[7] Boava LP , Cristofani-Yaly M, Machado MA. Physiologic, anatomic, and gene expression changes in *citrus sunki*, *Poncirus trifoliata*, and their hybrids after '*Candidatus* Liberibacter asiaticus' infection. Phytopathology. 2017;107:590–9 28068188 10.1094/PHYTO-02-16-0077-R

[8] Ma W , Pang Z, Huang X. et al. Citrus Huanglongbing is a pathogen-triggered immune disease that can be mitigated with antioxidants and gibberellin. Nat Commun. 2022;13:529 35082290 10.1038/s41467-022-28189-9 PMC8791970

[9] Xiong J , Wan X, Ran M. et al. Brassinosteroids positively regulate plant immunity via BRI1-EMS-SUPPRESSOR 1-mediated *GLUCAN SYNTHASE-LIKE 8* transcription. Front Plant Sci. 2022;13:854899 35401617 10.3389/fpls.2022.854899 PMC8988940

[10] Zou X , Bai X, Wen Q. et al. Comparative analysis of tolerant and susceptible citrus reveals the role of methyl salicylate signaling in the response to Huanglongbing. J Plant Growth Regul. 2019;38:1516–28

[11] Deng H , Achor D, Exteberria E. et al. Phloem regeneration is a mechanism for Huanglongbing-tolerance of ‘Bearss’ lemon and ‘LB8-9’ sugar belle® mandarin. Front Plant Sci. 2019;10:277 30949186 10.3389/fpls.2019.00277 PMC6435995

[12] Liu Y , Dong L, Ran D. et al. A comparative analysis of three rutaceae species reveals the multilayered mechanisms of citrus in response to Huanglongbing disease. J Plant Growth Regul. 2023;42:7564–79

[13] Zou X , Zhao K, Liu Y. et al. Overexpression of salicylic acid carboxyl methyltransferase (*CsSAMT1*) enhances tolerance to Huanglongbing disease in Wanjincheng orange (*Citrus sinensis* (L.) Osbeck). Int J Mol Sci. 2021;22:2803 33802058 10.3390/ijms22062803 PMC7999837

[14] Hong Z , Delauney AJ, Verma DP. A cell plate-specific callose synthase and its interaction with phragmoplastin. Plant Cell. 2001;13:755–68 11283334 10.1105/tpc.13.4.755 PMC135532

[15] Niu Q , Zhang P, Su S. et al. Characterization and expression analyses of Callose synthase enzyme (*Cals*) family genes in maize (*Zea mays* L.). Biochem Genet. 2022;60:351–69 34224040 10.1007/s10528-021-10103-5

[16] Feng J , Chen Y, Xiao X. et al. Genome-wide analysis of the CalS gene family in cotton reveals their potential roles in fiber development and responses to stress. PeerJ. 2021;9:e12557 34909280 10.7717/peerj.12557 PMC8641485

[17] Zhang Q , Wang Z, Qi J. et al. The advances of callose synthase in plant. Acta Horticulturae Sinica. 2021;48:661–75

[18] Ellinger D , Naumann M, Falter C. et al. Elevated early callose deposition results in complete penetration resistance to powdery mildew in Arabidopsis. Plant Physiol. 2013;161:1433–44 23335625 10.1104/pp.112.211011 PMC3585607

[19] Blümke A , Somerville SC, Voigt CA. Transient expression of the *Arabidopsis thaliana* callose synthase PMR4 increases penetration resistance to powdery mildew in barley. Adv Biosci Biotechnol. 2013;04:810–3

[20] Cheng P , Wang Z, Ren Y. et al. Silencing of a wheat ortholog of glucan synthase-like gene reduced resistance to *Blumeria graminis* f. sp. *tritici*. Front Plant Sci. 2021;12:800077 35003189 10.3389/fpls.2021.800077 PMC8735228

[21] Chowdhury J , Schober MS, Shirley NJ. et al. Down-regulation of the *glucan synthase-like 6* gene (*HvGsl6*) in barley leads to decreased callose accumulation and increased cell wall penetration by *Blumeria graminis* f. sp. *hordei*. New Phytol. 2016;212:434–43 27364233 10.1111/nph.14086

[22] Enrique R , Siciliano F, Favaro MA. et al. Novel demonstration of RNAi in citrus reveals importance of citrus callose synthase in defence against *Xanthomonas citri* subsp. *citri*. Plant Biotechnol J. 2011;9:394–407 20809929 10.1111/j.1467-7652.2010.00555.x

[23] Bernardini C , Santi S, Mian G. et al. Increased susceptibility to chrysanthemum yellows phytoplasma infection in Atcals7ko plants is accompanied by enhanced expression of carbohydrate transporters. Planta. 2022;256:43 35842878 10.1007/s00425-022-03954-8 PMC9288947

[24] Achor D , Welker S, Ben-Mahmoud S. et al. Dynamics of *Candidatus* Liberibacter asiaticus movement and sieve-pore plugging in citrus sink cells. Plant Physiol. 2020;182:882–91 31818905 10.1104/pp.19.01391 PMC6997701

[25] Granato LM , Galdeano DM, D’Alessandre NDR. et al. Callose synthase family genes plays an important role in the *citrus* defense response to *Candidatus* Liberibacter asiaticus. Eur J Plant Pathol. 2019;155:25–38

[26] Nishimura MT , Stein M, Hou BH. et al. Loss of a callose synthase results in salicylic acid-dependent disease resistance. Science. 2003;301:969–72 12920300 10.1126/science.1086716

[27] Dong X , Hong Z, Chatterjee J. et al. Expression of callose synthase genes and its connection with *Npr1* signaling pathway during pathogen infection. Planta. 2008;229:87–98 18807070 10.1007/s00425-008-0812-3

[28] Wang H , Cao S, Li T. et al. Classification and expression analysis of cucumber (*Cucumis sativus* L.) callose synthase (*CalS*) family genes and localization of CsCalS4, a protein involved in pollen development. Biotechnol Biotec Eq. 2021;35:1992–2004

[29] Mbiza NIT , Hu Z, Zhang H. et al. GhCalS5 is involved in cotton response to aphid attack through mediating callose formation. Front Plant Sci. 2022;13:892630 35937318 10.3389/fpls.2022.892630 PMC9350506

[30] Tomczynska I , Stumpe M, Doan TG. et al. A Phytophthora effector protein promotes symplastic cell-to-cell trafficking by physical interaction with plasmodesmata-localised callose synthases. New Phytol. 2020;227:1467–78 32396661 10.1111/nph.16653

[31] Shen P , Li XY, Fu SM. et al. A "Candidatus Liberibacter asiaticus"-secreted polypeptide suppresses plant immune responses in Nicotiana benthamiana and Citrus sinensis. Front Plant Sci. 2022;13:997825 36352861 10.3389/fpls.2022.997825 PMC9638108

[32] Pitino M , Armstrong CM, Cano LM. et al. Transient expression of *Candidatus* Liberibacter asiaticus effector induces cell death in *Nicotiana benthamiana*. Front Plant Sci. 2016;7:982 27458468 10.3389/fpls.2016.00982 PMC4933711

[33] Liu JL , Du H, Ding X. et al. Mechanisms of callose deposition in rice regulated by exogenous abscisic acid and its involvement in rice resistance to *Nilaparvata lugens* Stal (Hemiptera: Delphacidae). Pest Manag Sci. 2017;73:2559–68 28664567 10.1002/ps.4655

[34] Singh RK , Miskolczi P, Maurya JP. et al. A tree ortholog of SHORT VEGETATIVE PHASE floral repressor mediates photoperiodic control of bud dormancy. Curr Biol. 2019;29:128–133.e2 30554900 10.1016/j.cub.2018.11.006

[35] Tylewicz S , Petterle A, Marttila S. et al. Photoperiodic control of seasonal growth is mediated by ABA acting on cell-cell communication. Science. 2018;360:212–5 29519919 10.1126/science.aan8576

[36] Rosales R , Burns JK. Phytohormone changes and carbohydrate status in sweet orange fruit from Huanglongbing-infected trees. J Plant Growth Regul. 2011;30:312–21

[37] Nehela Y , Hijaz F, Elzaawely AA. et al. Citrus phytohormonal response to *Candidatus* Liberibacter asiaticus and its vector *Diaphorina citri*. Physiol Mol Plant Pathol. 2018;102:24–35

[38] Flors V , Ton J, Jakab G. et al. Abscisic acid and callose: team players in defence against pathogens? J Phytopathol. 2005;153:377–83

[39] Skubacz A , Daszkowska-Golec A, Szarejko I. The role and regulation of ABI5 (ABA-insensitive 5) in plant development, abiotic stress responses and phytohormone crosstalk. Front Plant Sci. 2016;7:1884 28018412 10.3389/fpls.2016.01884 PMC5159420

[40] Dong T , Park Y, Hwang I. Abscisic acid: biosynthesis, inactivation, homoeostasis and signalling. Essays Biochem. 2015;58:29–48 26374885 10.1042/bse0580029

[41] Zhao H , Nie K, Zhou H. et al. ABI5 modulates seed germination via feedback regulation of the expression of the PYR/PYL/RCAR ABA receptor genes. New Phytol. 2020;228:596–608 32473058 10.1111/nph.16713

[42] Collin A , Daszkowska-Golec A, Szarejko I. Updates on the role of ABSCISIC ACID INSENSITIVE 5 (ABI5) and ABSCISIC ACID-RESPONSIVE ELEMENT BINDING FACTORs (ABFs) in ABA signaling in different developmental stages in plants. Cells. 2021;10:1996.c 34440762 10.3390/cells10081996 PMC8394461

[43] Wu L , Bai X, Wen Q. et al. Early spread characteristics of *Candidatus* Liberibacter asiaticus in Jincheng orange (*Citrus sinensis* Osbeck) by leafdisc grafting. Acta Horticulturae Sinica. 2018;45:2121–8

[44] Chen C , Chen H, Zhang Y. et al. TBtools: an integrative toolkit developed for interactive analyses of big biological data. Mol Plant. 2020;13:1194–202 32585190 10.1016/j.molp.2020.06.009

[45] Tamura K , Stecher G, Kumar S. MEGA11: molecular evolutionary genetics analysis version 11. Mol Biol Evol. 2021;38:3022–7 33892491 10.1093/molbev/msab120 PMC8233496

[46] Yang W , Fu J, Huang X. et al. Identification of the citrus superoxide dismutase family and their roles in response to phytohormones and citrus bacterial canker. Agriculture. 2022;12:1254

[47] Yao L , Fan H, Zhang Q. et al. Function of citrus bacterial canker resistance-related transcription factor CitMYB20. Sci Agric Sin. 2020;53:1997–2008

[48] Lescot M , Déhais P, Thijs G. et al. PlantCARE, a database of plant cis-acting regulatory elements and a portal to tools for in silico analysis of promoter sequences. Nucleic Acids Res. 2002;30:325–7 11752327 10.1093/nar/30.1.325 PMC99092

[49] Zou L , Liu W, Zhang Z. et al. Gene body demethylation increases expression and is associated with self-pruning during grape genome duplication. Hortic Res. 2020;7:84 32528696 10.1038/s41438-020-0303-7 PMC7261773

[50] Li Q , Qin X, Zhang M. et al. CsBZIP40 confers resistance against citrus bacterial canker by repressing CsWRKY43-CsPrx53/CsSOD13 cascade mediated ROS scavenging. Hortic Res. 2023;10:uhad138 37575655 10.1093/hr/uhad138 PMC10421728

[51] Li F , Dai SM, Deng ZN. et al. Evaluation of parameters affecting *Agrobacterium*-mediated transient expression in citrus. J Integr Agric. 2017;16:572–9

[52] Acanda Y , Welker S, Orbović V. et al. A simple and efficient agroinfiltration method for transient gene expression in *citrus*. Plant Cell Rep. 2021;40:1171–9 33948685 10.1007/s00299-021-02700-w

