# Supplementary figures and images for: ABA-CsABI5-CsCalS11 module upregulates Callose deposition of citrus infected with Candidatus Liberibacter asiaticus
Source: Hortic Res. 2023 Dec 23;11(2):uhad276. doi: 10.1093/hr/uhad276 (PMC10857934; doi:10.1093/hr/uhad276)

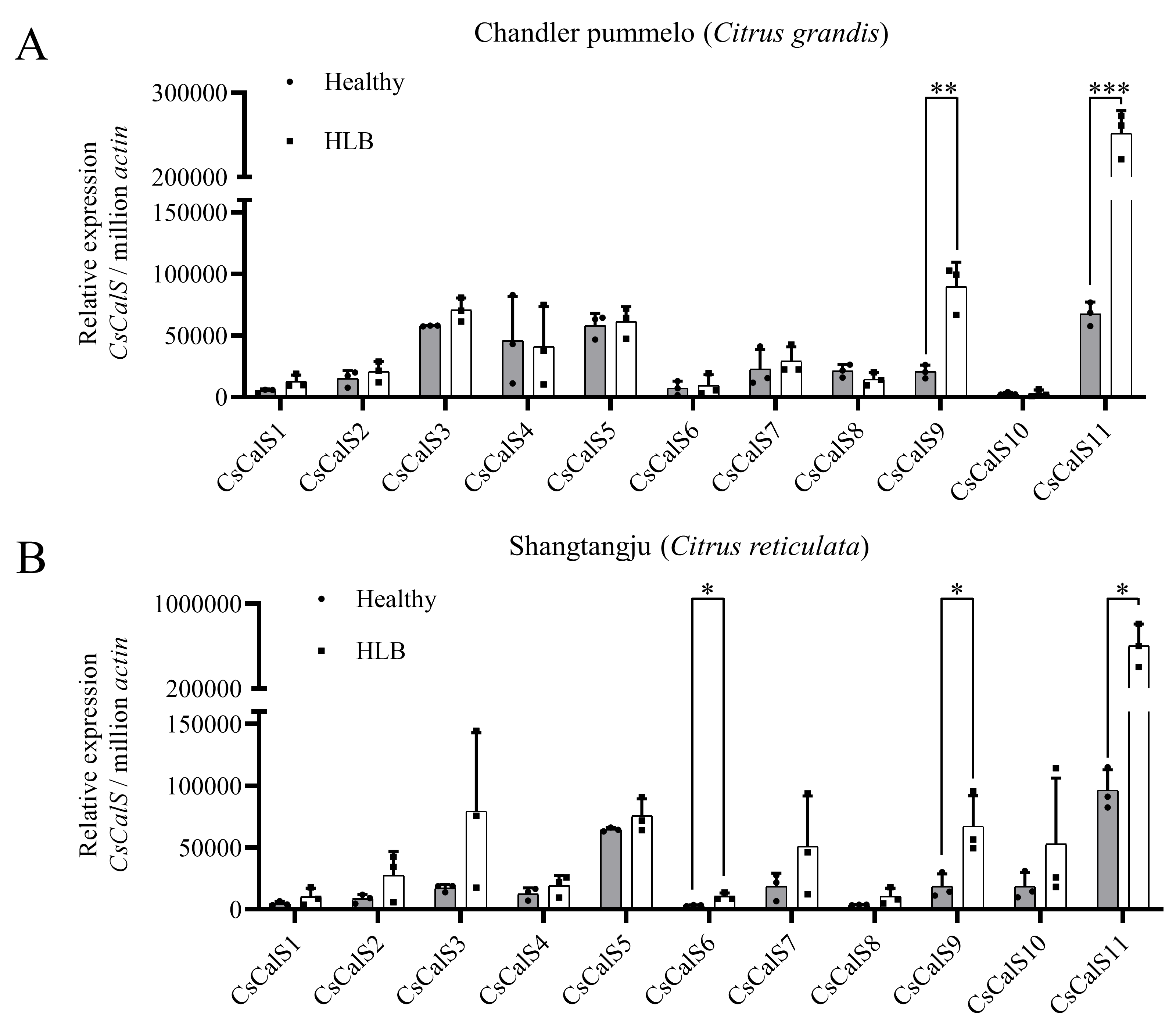

Supplement: Web_Material_uhad276 [file web_material_uhad276.zip › Supplemental Fig 1.tif]

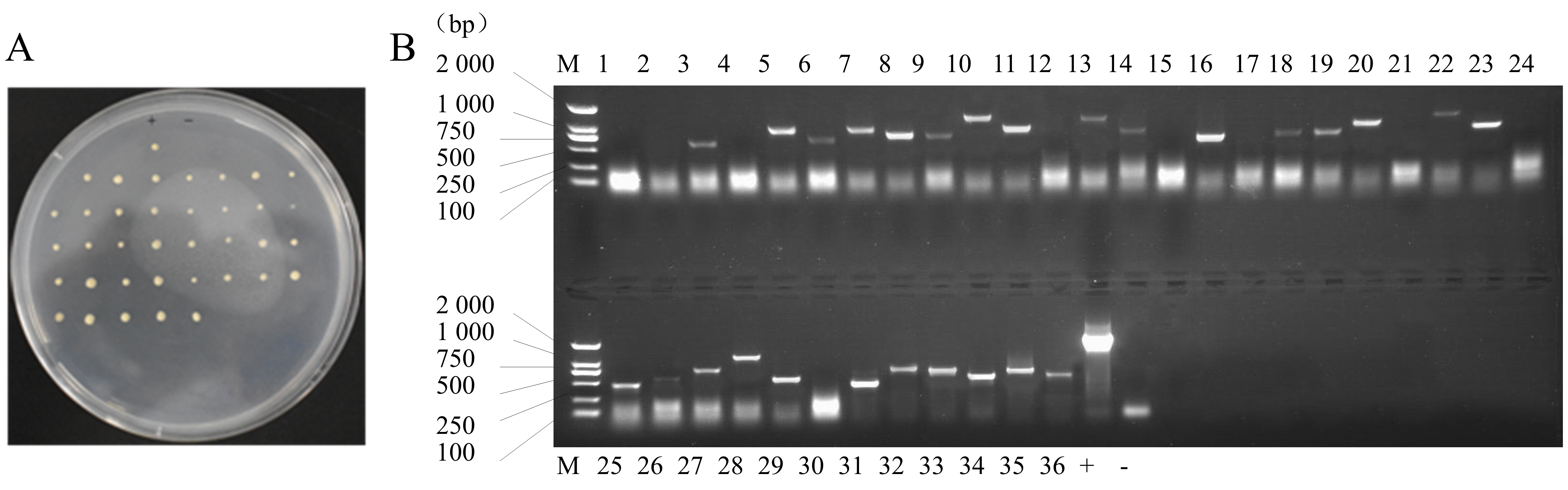

Supplement: Web_Material_uhad276 [file web_material_uhad276.zip › Supplemental Fig 2.tif]
